# Progress in Surgery

**Published:** 1894-05-05

**Authors:** 


### PROGRESS IN SURGERY.

### THE SURGICAL TREATMENT OF PULMONARY CAVITIES AND LOCALISED AREAS OF TUBERCULOUS LUNGS.

The question whether it is wise to open and drain tuberculous pulmonary cavities is discussed by Dr. Arnold Chaplin in the *Practitioner*.[1] He considers that it is almost impossible to tell how much the symptoms present are due to the existence of the cavity, and how much to active tuberculous processes going on in the lung tissue around, and hence that we may often fail to relieve by draining the cavity. The hectic temperature, for instance, which in an abscess elsewhere of non-tuberculous nature we should regard as an urgent indication for free drainage, in the case of a tuberculous lung cavity may be due, not to any septic absorption, but to disseminated tuberculosis in other parts of the lung, of which even physical signs do not give us any indication. Yet although Dr. Chaplin lays so much stress on the fact that widely disseminated tuberculosis, and not the retention of pus in the cavity, is so commonly the cause of the symptoms, we must remember that in some cases in which tuberculous cavities have been drained the hectic temperature has at once ceased, and recurred if the opening closed.

Dr. Chaplin considers a basic cavity much more suitable for drainage than an apical one. In a basic cavity expectoration is more difficult, and the tuberculosis is more likely to be of a limited character. Moreover, the cavity is easily reached and very satisfactorily drained. The impossibility of being certain that the physical signs are due to one large cavity rather than several smaller ones close together, or even in some cases whether excavation or consolidation is present, is referred to in this paper. The presence of adhesions between the two pleural surfaces over the cavity are also mentioned as favourable to operation, but this, of course, can only be determined at the time of operation, or by inserting a needle into the lungs and observing if it moved up and down with the lung, which would indicate the absence of pleural adhesions. Dr. Chaplin says if adhesions are absent it is impossible to prevent the pus entering the pleural cavity, but he does not seem to be familiar with the method of suturing the two surfaces of pleura together before opening the lung abscess. A case is referred to in which there were physical signs of cavity at the base of one lung, with profuse fœtid expectoration and hectic temperature and emaciation. Bronchiectasis was diagnosed, and the cavity opened, but the patient died in a few days, and general tuberculosis of the other lung was discovered, which had not been revealed by a careful physical examination; and, moreover, the cavity opened was found to be only one of many dilated bronchial tubes. This case well illustrates some of the difficulties in the diagnosis of pulmonary conditions which may be treated surgically.

The question as to the advisability of excising tuberculous areas of lung is also discussed in Dr. Chaplin's paper, and he points out the impossibility of ascertaining by physical signs the extent of the lesion, and whether or not it is present in the other lung as well. If it is central, or if widely disseminated, what good can operation do, and yet who shall say in any given case that it is not?

The surgical treatment of pulmonary cavities is very ably discussed by Dr. Pendleton Dandridge, of Cincinnati, in the *Annals of Surgery*.[2] He thinks we ought to drain large tubercular cavities because of the septic absorption going on from them, even though they are associated with processing tuberculous disease in the lung. He asks: "What surgeon would hesitate to drain or scrape out tuberculous abscesses in other parts of the body, because there happened to be evidence of commencing disease in the lungs or tubercular foci elsewhere?" But the difficulty seems to be, as Dr. Chaplin points out in the paper just referred to, that the hectic temperature which would be the chief sign of septic absorption, may on the other hand be due to advancing tuberculosis. But perhaps we shall do well to give the patient the benefit of the doubt, and drain in suitable cases. Dr. Dandridge agrees with Dr. Chaplin that a suitable case is a basic rather than an apex cavity, because expectoration of the contents of the former is more difficult, and it is more easily reached with regard to bronchiectatic cavities. Dr. Dandridge agrees that in most of the cases the dilatation is not confined to a single tube, but he thinks that at times some cavities are so much larger than others that they may with great advantage be drained, as they furnish by far the greatest amount of putrid secretion.

Large abscesses, non-tuberculous, and sometimes due to aspiration forms of pneumonia; abscess from gangrene; and hydatid cysts should, he considers, always be drained. The question as to the presence of adhesion between the two surfaces of pleura over the cavity is carefully considered. This is said to be more likely to have taken place in tuberculous cavities than in bronchiectatic ones, which are more deeply situated as a rule. It is maintained that the question of adhesions can be readily settled, unless the lung be consolidated, by inserting an aseptic needle, when if no adhesion has taken place the point will move as the lung moves. But the question of consolidation around a tuberculous cavity is a very difficult one to diagnose. He thinks Godler's plan of stitching the two surfaces together if not adherent is not only very difficult, but very unsatisfactory, as cases are reported in which an empyema has followed the opening of a lung abscess after such suturing had been carried out. He would be inclined to delay opening the cavity, and packing with gauze for a few days, rather than suturing. Perhaps a better plan would be to suture, and then wait for a few days. In one case in which we sutured, the suture tore out of the pleura as we pushed the trochar into the lung. Dr. Dandridge advises the use of the cautery in cutting through the lung into the cavity, and it is certainly not likely to break down slight adhesion, as a plunge with a trochar and cannula, as recommended by Godler would be.

The operation for draining tuberculous apex cavities as minutely described by Poirier and Jounesco, is also given in Dr. Dandridge's paper.

Dr. Priestley Leech[3] reports a case of gangrenous abscess of the lung, drained, with recovery. The patient was a young man æt. 22. He had a three weeks' illness in April, beginning with painful breathing, pneumonia, and in June coughed up a large quantity of very offensive sputum. The cough and expectoration continued, and in September he consulted Dr. Priestley Leech, who detected an area of dulness with diminished breath sounds and vocal resonance over the middle of the left lung, back and front. Although the physical signs were not those of a large cavity, the symptoms and localisation of the lesion in the lung suggested one, and on October 8th Dr. Leech operated. Through the first incision the dull area was explored six times without finding pus. This was then sewn up and another incision made, through which the second introduction of the needle found some horribly offensive pus. The pleura was found adherent, and the abscess opened by passing Lister's sinus forceps along the needle. During the administration of chloroform the patient coughed up some pus, and no danger arose from retention of pus in the bronchial tubes. The cavity continued to discharge for six months. During this time he had pneumonia in the affected lung, and once an attack of rather serious hæmoptysis. Since the cessation of the discharge he had remained in good health. Dr. Leech thinks that the reason the patient took so long to recover was because a loculus of the abscess cavity was probably situated at a lower level than the opening of the drainage tube. He points out the need for many punctures in some cases before the pus is found. It is recommended that the patient should empty his cavity by blowing at intervals with his mouth and nostrils closed. Dr. Leech considers operation is not justifiable unless the patient is losing ground, and in support of this view relates a case of recovery from lung abscess. The physical signs were in the upper part of the left lung, but apart from their position they might have been due to localised empyæma communicating with the bronchus.

[1] Practitioner, Jan., 1894. [2] Annals of Surgery, Feb., 1894. [3] Lancet, Jan. 13, 1894.

